# Alternative tissue fixation for combined histopathological and molecular analysis in a clinically representative setting

**DOI:** 10.1007/s00418-021-02029-1

**Published:** 2021-12-14

**Authors:** Amelia Meecham, Elena Miranda, Hayley T. Morris, Jane Hair, Karin A. Oien, Gareth Gerrard, Naomi Guppy, David Mooney, Emily C. Shaw, Margaret Ashton-Key, Robert Lees, Adrienne Flanagan, Manuel Rodriguez-Justo

**Affiliations:** 1 Research Department of Pathology, UCL Cancer Institute, London, UK; 2 Institute of Cancer Sciences, University of Glasgow, Glasgow, UK; 3 Department of Cellular Pathology, University Hospital Southampton NHS Foundation Trust, Southampton, UK; 4 Cancer Sciences Unit, University of Southampton, Southampton, UK; 5 Royal National Orthopaedic Hospital NHS Trust, Stanmore, Middlesex UK; 6 NHS Research Scotland Greater Glasgow and Clyde Biorepository, Glasgow, UK; 7 Department of Pathology, Queen Elizabeth University Hospital, Glasgow, UK; 8 University College Hospitals, London, UK; 9 Sarah Cannon Molecular Diagnostics, HCA Healthcare UK, London, UK

**Keywords:** Formalin, DNA, RNA, PAXgene, Fixative, FFPE

## Abstract

Formalin is the principal tissue fixative used worldwide for clinical and research purposes. Despite optimal preservation of morphology, its preservation of DNA and RNA is poor. As clinical diagnostics increasingly incorporates molecular-based analysis, the requirement for maintaining nucleic acid quality is of increasing importance. Here we assess an alternative non-formalin-based tissue fixation method, PAXgene Tissue system, with the aim of better preserving nucleic acids, while maintaining the quality of the tissue to be used for vital existing diagnostic techniques. In this study, these criteria are assessed in a clinically representative setting. In total, 203 paired PAXgene Tissue and formalin-fixed samples were obtained. Blind-scored haematoxylin and eosin (H&E) sections showed comparable and acceptable staining. Immunohistochemistry (IHC) staining was suboptimal using existing protocols but improved with minor method adjustment and optimisation. Quality of DNA and RNA was significantly improved by PAXgene tissue fixation [RIN 2.8 versus 3.8 (*p* < 0.01), DIN 5.68 versus 6.77 (*p* < 0.001)], which translated into improved performance on qPCR assay. These results demonstrate the potential of PAXgene Tissue to be used routinely in place of formalin, maintaining adequate histological staining and significantly improving the preservation of biological molecules in the genomic era.

**Supplementary Information:**

The online version contains supplementary material available at 10.1007/s00418-021-02029-1.

## Introduction

Formalin-fixed paraffin-embedded (FFPE) tissue samples are the foundation of pathology services and are regarded as essential and irreplaceable for diagnostic purposes. This is despite formalin’s status as a category 1B carcinogen (European Commission regulation 605/2014) and damaging effect on biomolecules such as DNA and RNA. Formalin fixes tissue by creating intra- and inter-molecular cross-links, primarily between lysines (Thavarajah et al. 2012). Cross-links also occur between histones and directly with nucleotides (Brutlag et al. 1969; McGhee and von Hippel 1975a, b). The downstream effects of cross-linking for nucleic acids is degradation (Impraim et al., 1987), making them more difficult to extract and potentially altering sequences (Williams et al., 1999). As clinical diagnostics is moving towards more regular use of molecular techniques, the substandard quality of nucleic acids available from FFPE tissue has become an increasingly recognised issue in pathology.

PAXgene Tissue is a non-carcinogenic, alcohol-based fixative developed by PreAnalytix, a collaboration between QIAGEN and BD (Becton, Dickindon and Company). Since alcohol-based fixatives do not form the damaging cross-links observed in FFPE, PAXgene-fixed paraffin-embedded (PFPE) tissue should allow for traditional histopathology methods, including morphology and immunohistochemistry (IHC), to be performed alongside newer molecular diagnostic techniques [e.g. fluorescent in situ hybridisation (FISH) and DNA sequencing], by better preserving biological molecules.

The PAXgene Tissue fixation system requires specimen immersion in an alcohol-based initial fixative solution (for 3–24 h) followed by transfer to a subsequent stabiliser solution. The stabiliser allows for storage of the sample up to 7 days at room temperature (15–25 °C) or up to 4 weeks at 2–8 °C, with longer-term storage options including sample freezing also reviewed (Groelz et al. 2018). The recommended fixation time when using formalin, however, is more limited (usually 24–48 h). In the clinical setting, samples are routinely formalin fixed over the weekend, which is considered acceptable for the purposes of current diagnostic techniques (H&E, IHC and FISH), but over-fixation is particularly detrimental to the preservation of nucleic acids (Miething et al., 2006).

PAXgene Tissue’s preservation of tissue morphology has been well studied (Belloni et al. 2013; Kap et al. 2011; Viertler et al. 2012; Groelz et al. 2013), providing evidence through blinded scoring of H&E-stained slides that PFPE morphology is comparable to that achieved on FFPE, with some well-recognised artefacts such as hypereosinophilia of the tissue, and changes in erythrocyte appearance that do not hamper diagnostic assessment. Although these artefacts are of course important, the potential diagnostic value of high-quality molecular material available from fixed tissue is enormous.

Considerable work has also been performed to optimise FFPE-based IHC and FISH protocols for PFPE samples (Mathieson et al. 2016; Oberauner-Wappis et al. 2016) where minor changes to protocols result in comparable scoring. Achieving equivalence in morphological preservation and IHC is extremely important, as these are the cornerstone of clinical diagnosis as well as predictive immunohistochemical marker assessment (such as HER2, PD-L1 and ALK) used to inform patient management and treatment options,

STRATFix is a UK-based, Innovate UK funded clinical and academic consortium working in conjunction with QIAGEN. The objective of the study is to assess the implementation and performance of PAXgene Tissue in a routine cellular pathology workflow and environment, collecting a range of tissue types processed alongside routine samples, and evaluating these for morphology, IHC and molecular analysis, while also considering the practicalities of a new workflow for PAXgene tissue. Here we present the results of three sites across the UK.

## Materials and methods

### Sample collection

This project was approved by the UK National Research Ethics Service (NRES, Research Ethics Committee reference: 15/YH/0221). Participation through the UCL/UCLH Biobank was also approved by NRES (REC reference 15/YH/0311, HTA licence 12055 number) and the UCL/UCLH Biobank Ethics Committee (reference no. EC17.14).

Identically sized pairs (as close as possible to 5 mm^3^) of tumour tissue samples surplus to diagnostic requirements were collected from fresh tumour resection specimens received at each site, with one immediately placed in neutral buffered formalin (Genta Medical, York, UK) or PAXgene Tissue fixative (QIAGEN, Manchester, UK). Formalin samples were fixed for a maximum of 24 h, processed overnight (Excelsior AS Processor, Thermo Fisher Scientific, Cheshire, UK) and embedded in paraffin. The protocol consists of stepwise dehydration in 70%, 90%, 100% ethanol, followed by xylene and wax. PAXgene samples were fixed for a maximum of 24 h before being transferred to PAXgene stabiliser solution (QIAGEN, Manchester, UK). PAXgene samples were processed overnight in a formalin-free processor and paraffin-embedded in low-melting-point paraffin wax. Samples in fixative were stored at 4 °C, and all paraffin-embedded blocks were stored at −20 °C. The different tissues collected in the three different sites are listed in Table 1.

**Table 1:** Sample collection by tissue type and site

Tissue type	Number of cases			
	Site 1	Site 2	Site 3	Total
Colorectal	25	1	35	61
Prostate	43	0	0	43
Lung	1	0	31	32
Lymph node	2	0	17	19
Breast	0	3	9	12
Bladder	0	0	9	9
Kidney	0	2	5	7
Sarcoma	0	6	0	6
Oesophagus	3	0	0	3
Stomach	0	3	0	3
Pleura	0	0	2	2
Ovary	1	1	0	2
Thymus	0	0	1	1
Pancreas	0	0	1	1
Skin	0	0	1	1
Spleen	0	0	1	1
Total	75	16	112	203

In total, 203 paired samples were collected from three sites across the UK. The largest collections of tissue types obtained were colorectal (61 paired), prostate (43 paired cases) and lung (32 paired cases)

### Morphology

Immediately prior to sectioning, FFPE and PAXgene blocks were removed from the fridge or freezer, respectively, and 4 μm sections cut on a rotary microtome, stained with H&E and coverslipped (Multistainer, Leica, Milton Keynes, UK). Using a previously published scoring system, nuclear, cytoplasmic and cell membrane features were each assigned a score of 0–4 by two blinded pathologist observers (see Southwood et al. 2020 for details).

### Immunohistochemistry

Immunohistochemistry was performed relevant to each tissue type (see Supplementary Materials Table S1 for a full list of antibodies used). Antibodies were used as per manufacturer’s instructions on existing laboratory platforms for FFPE tissue in the first instance, enabling identification of those antibodies which would need optimisation for PFPE. For antibodies requiring additional optimisation, a pre-treatment consisting of incubation of the slides in 10% formalin buffered for 24 h before staining was performed. All antibodies (other than HER2) were applied following on-board heat-induced epitope retrieval (HIER) using Leica ER2 (high-pH epitope retrieval solution, pH9; cat. no. AR9640, Leica Biosystem).

### FISH

FISH was performed using SS18 break-apart probe (Abbott Molecular, Maidenhead Berkshire, UK), and carried out as described previously (Amary et al. 2014). PFPE sections were incubated for 24 h in formalin as per recommendations (Oberauner-Wappis et al. 2016). Deparaffinised sections were pre-treated by pressure cooking and incubated in pepsin solution at 37 °C for 50 min. Probes were added to tissue sections and denatured at 72 °C and hybridised overnight at 37 °C. Following hybridisation, the sections were washed and counterstained with 4′, 6-diamidino-2-phenylindole (DAPI) and mounted with coverslips.

### Nucleic acid extraction and analysis

DNA and RNA was extracted from 65 paired cases (FFPE and PFPE) using AllPrep DNA/RNA FFPE Kit (cat. no. 80234, QIAGEN, Manchester, UK) and PAXgene Tissue DNA Kit (cat. no. 767134, QIAGEN, Manchester, UK)/PAXgene Tissue RNA Kit (cat. no. 765134, QIAGEN, Manchester, UK), respectively. Four 10 µM sections of each paraffin embedded block were taken, and nucleic acids extracted following manufacturer’s instructions.

DNA and RNA concentrations were estimated using Thermo Fisher Scientific Qubit3 Fluorometric quantification system as per manufacturer’s instructions and yield calculated accordingly. Purity was estimated using Nanodrop 1000 spectrophotometer and fragmentation and DNA/RNA integrity numbers using Agilent 2200 Tapestation (as per manufacturer’s instructions). Qualitative PCR using Illumina Infinium FFPE QC kit (cat. no. WG-321-1001, Illumina, Cambridge, UK) was performed on DNA pairs with sufficient yield. RT-qPCR and subsequent gene expression analysis (TaqMan assay, Thermo Fisher Scientific, Cheshire, UK) of Glucuronidase Beta (GUSB) was performed on a subset of RNA pairs (*n* = 19). All qPCR was performed in triplicate.

### Sequencing

Four cases with identical FFPE, PFPE and FF samples were sequenced by Sarah Cannon Molecular Diagnostics for somatic variant analysis.

#### Library preparation and templating

In total, 10 ng of DNA from each sample, along with internal quality control (IQC) material (HD732/HD733; Horizon Discovery, Cambridge, UK) and a no template control (NTC), was used to prepare barcoded Ion Torrent libraries using the 50-gene Ampliseq Cancer Hotspot Panel v2 (CHPv2) primers and Oncomine Solid Tumour Library Prep Kit (Life Technologies, Carlsbad, CA, USA). The libraries were made equimolar prior to pooling using the in-kit Equalizer beads. The emulsion PCR and ion sphere particle (ISP) templating of the pooled libraries was performed using the One Touch 2 platform and templated ISP enrichment using the One Touch ES system (Life Technologies, Thermo Fisher Scientific, Cheshire, UK).

#### NGS and informatics

The final templated library pool was loaded onto a 318v2 sequencing chip and sequenced on an Ion Torrent Personal Genome Machine (PGM) (Life Technologies, Thermo Fisher Scientific, Cheshire, UK) using a 500flow template. The base calling, alignment (to human genome build hg19/GRCh37) and assembly were performed using the on-board Torrent Suite v5.05 software, and variant calling by the associated variant Caller plug-in, using the CHPv2 region file and a custom hot-spot Browser Extensible Data (BED) derived from COSMIC v79 (Catalogue Of Somatic Mutations In Cancer database; http://www.cancer.sanger.ac.uk).

## Results

### H&E morphology

To investigate the conservation of morphological characteristics under PAXgene fixation, H&E staining of PFPE and FFPE specimens was evaluated in parallel. In total, 203 paired formalin- and PAXgene-fixed samples were collected from three UK sites. These comprised 16 different tissue types, which are summarised in Table 1.

**Fig. 1:**
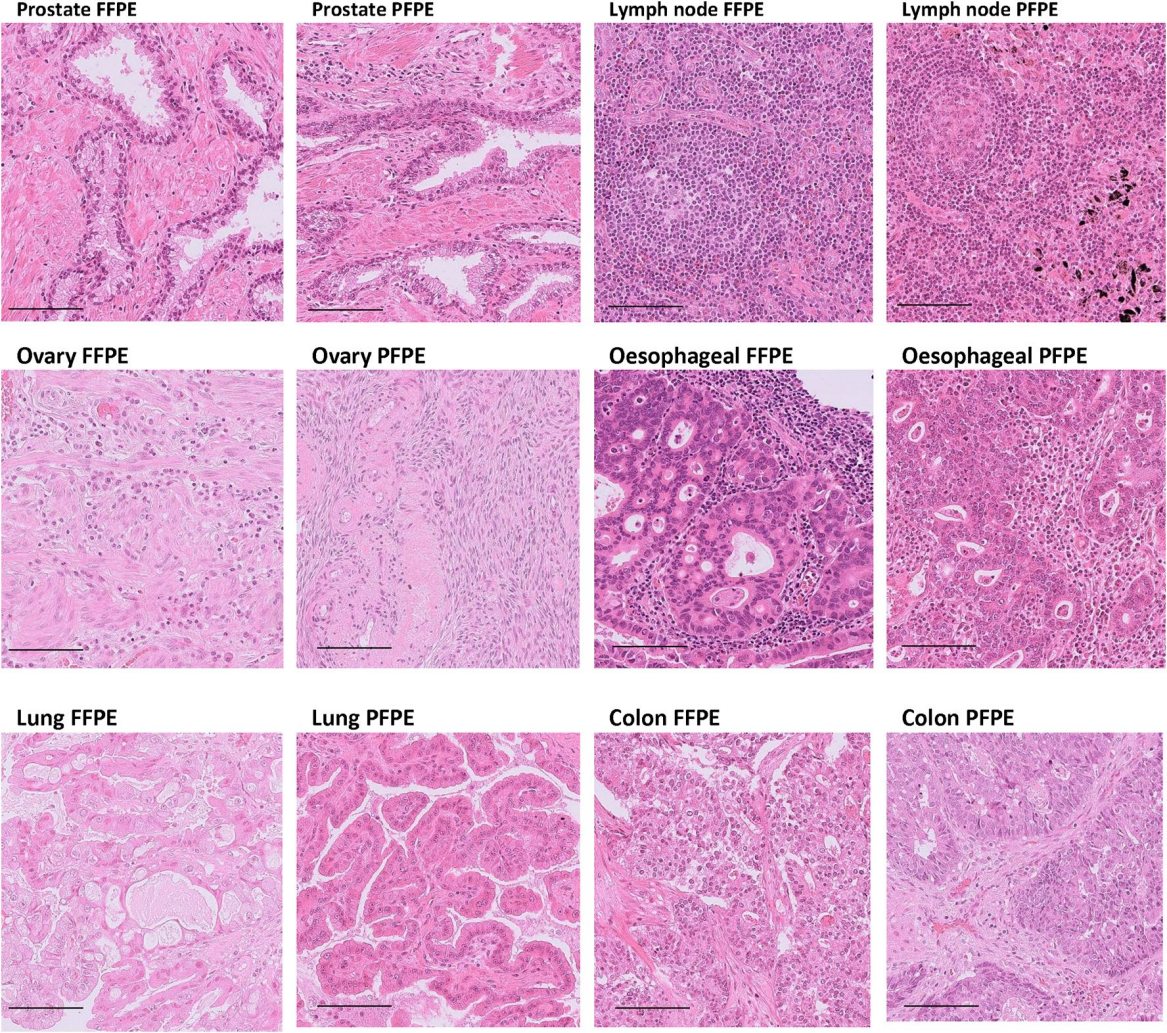
Representative H&E-stained sections from formalin- and PAXgene-fixed tissues. Images presented are from a range of tissue types and demonstrate the hypereosinophilia observed in the PAXgene-fixed tissues. Scoring of the quality of these sections can be found in Table 2. Scale bars represent 100 µm

Blinded morphological scoring of 180 FFPE/PFPE pairs revealed no statistically significant difference in membrane and nuclear staining; however, scoring of cytoplasmic staining was lower in PFPE samples (*p* = 0.0323 mean FFPE 3.633 + 0.578 versus PFPE 3.508 + 0.524) (Table 2). No significant differences were found in the overall scores. Representative H&E images can be found in Fig. 1, and analysis summarised in Table 2.

**Table 2:** H&E analysis

Site	Fixative	Number of cases	Morphological assessment	*p*	Nuclear	*p*	Cytoplasm	*p*	Cell membrane	*p*
Site 1	FFPE	70	10.49 + 1.77	0.0633	3.53 + 0.65	0.0061	3.49 + 0.65	ns	3.47 + 0.68	ns
	PAXgene	70	10.96 + 1.15		3.79 + 0.41		3.54 + 0.53		3.63 + 0.49	
Site 2	FFPE	16	7.5 + 1.46	0.0002	2.44 + 0.73	0.0027	2.75 + 0.58	0.0061	2.31 + 0.6	0.0011
	PAXgene	16	9.81 + 1.56		3.38 + 0.89		3.38 + 0.62		3.06 + 0.57	
Site 3	FFPE	94	11.681 + 0.469	0.0001	3.920 + 0.184	0.0001	3.894 + 0.23	0.0001	3.867 + 0.222	0.0029
	PAXgene	94	10.798 + 1.210		3.58 + 0.448		3.505 + 0.505		3.713 + 0.443	
Total	FFPE	180	10.844 + 1.707	ns	3.636 + 0.634	ns	3.633 + 0.578	0.0323	3.575 + 0.65	ns
	PAXgene	180	10.772 + 1.252		3.642 + 0.502		3.508 + 0.524		3.622 + 0.503	

H&E sections were blindly scored by two independent qualified observers, scoring each cellular component from 0 to 4, for a total maximum score of 12. Data represent mean score + SD. A paired Student’s *t*-test was performed to determine significance

Considering the three sites independently, the results varied. In site 1, overall scores showed no significant differences, while the nuclear staining was statistically improved in PFPE samples (PFPE 3.79 + 0.41 versus FFPE 3.53 + 0.65, *p* = 0.0061). Site 2 observers preferred nuclear, cytoplasm and membrane staining in the PFPE samples (3.38 + 0.89, 3.38 + 0.62, 3.06 + 0.57 respectively) compared with the FFPE samples (2.44 + 0.73, 2.75 + 0.58, 2.31 + 0.6, respectively; *p* ≤ 0.0061). Site 3 observers significantly preferred nuclear, cytoplasm and membrane staining of FFPE samples (3.92 + 0.184, 3.894 + 0.23, 0.867 + 0.222) compared with PFPE samples (3.58 + 0.448, 3.505 + 0.505, 3.713 + 0.443; *p* ≤ 0.0029).

A number of the pathologists, particularly in site 3, reported that PFPE sections could be identified easily due to a generalised increased intensity of eosin staining in the section and also swelling and central clearing of erythrocytes, both recognised artefacts in other studies using PAXgene Tissue system (Kap et al. 2011; Groelz et al. 2013). There were signs of increased tissue fragility in PFPE tissue compared with FFPE tissue, particularly in necrotic areas where tearing of sections was more commonly seen. Lymph nodes showed noticeably inferior preservation in PAXgene Tissue, with cell shrinkage and tissue disaggregation as well as slightly less crisp nuclear features.

When considering the different tissue types, the results reflected the general trend for the three different sites. For colon samples, site 1 preferred PFPE (*p* = 0.05) but site 3 preferred FFPE samples (*p* < 0.0001). For lymph nodes, lung and kidney site 3 preferred FFPE samples (*p* = 0.002, *p* = 0.0041 and *p* = 0.0372 respectively). For sarcoma, breast and stomach, site 2 preferred PFPE samples compared with FFPE samples (*p* = 0.0385, *p* = 0.0352 and *p* = 0.0003, respectively). Full breakdown of scoring from each site by tissue type can be found in Supplementary Materials (Table S2).

### Immunohistochemistry and FISH

**Fig. 2:**
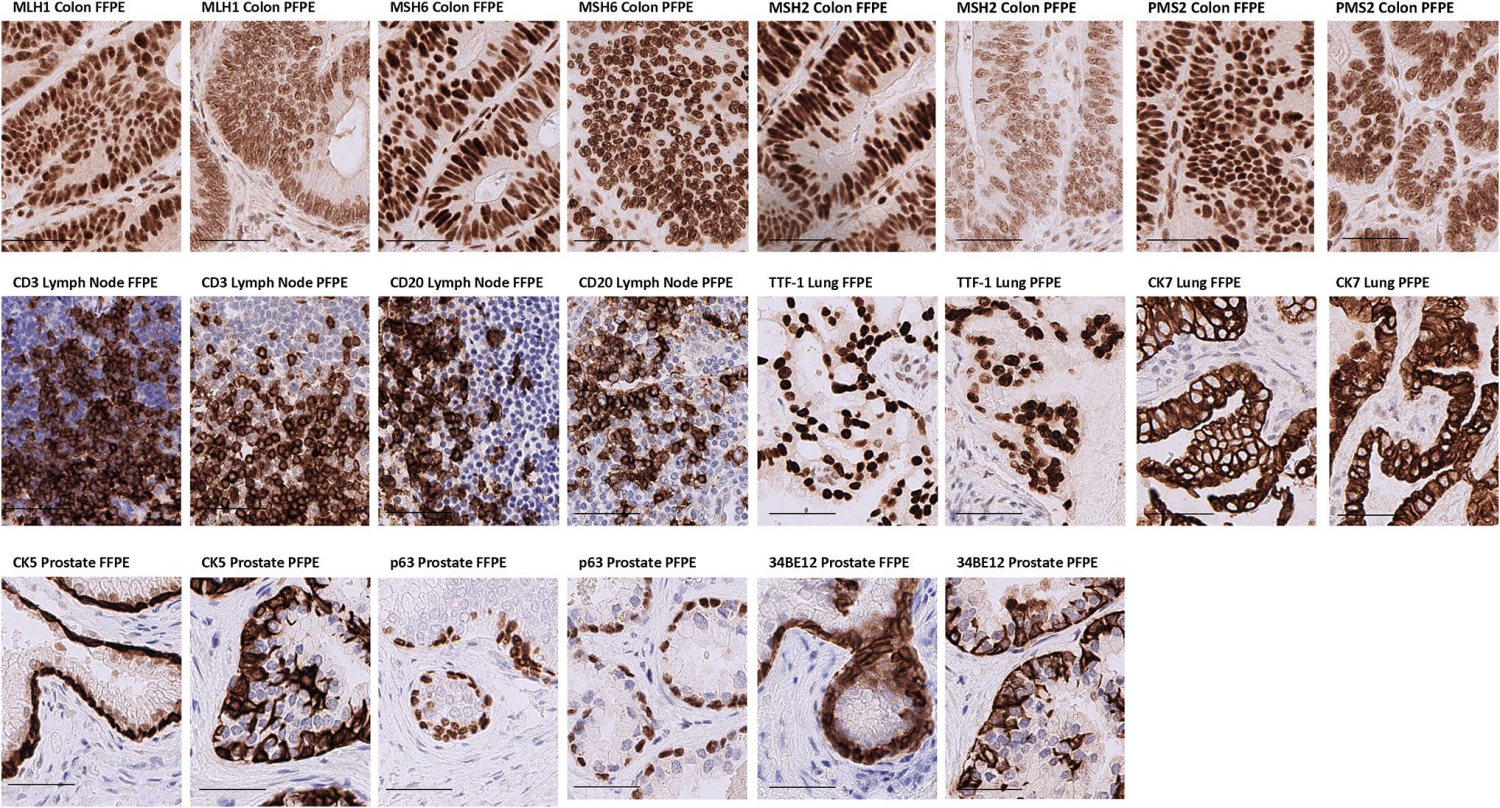
IHC staining. Representative images of IHC staining performed on paired FFPE and PFPE samples using MLH1, MSH6, MSH2, PMS2, CD3, CD20, TIF-1, CK7, CK5, p53 and HMWCK (34BE12) antibodies. Staining was performed using pre-optimised conditions for FFPE tissues. Scale bars represent 50 µm

For the purpose of comparing protein staining between PFPE and FFPE tissue, IHC for different antibodies was performed using protocols generated originally for FFPE samples. The results varied between the different sites and by tissue type (Table 3). Representative images can be found in Fig. 2.

**Table 3:** Analysis of IHC scoring

Tissue	Antibody	Number of cases	Fixative	Mean + SD	*p*-Value
Colon	MLH1	18	FFPE	4.394 + 0.362	0.001
			PFPE	3.778 + 0.628	
	MSH2	18	FFPE	4.483 + 0.368	0.0001
			PFPE	3.717 + 0.538	
	PMS2	18	FFPE	4.383 + 0.268	0.0022
			PFPE	3.950 + 0.484	
	MSH6	18	FFPE	4.7 + 0.34	0.0276
			PFPE	4.367 + 0.512	
	p53	18	FFPE	4.089 + 0.721	0.0736
			PFPE	3.633 + 0.759	
	p16	8	FFPE	4.063 + 0.177	0.2011
			PFPE	3.875 + 0.354	
	EGFR	6	FFPE	3.517 + 0.354	0.835
			PFPE	3.571 + 0.535	
Lung	P63	8	FFPE	3.438 + 1.613	0.002
			PFPE	1.250 + 0.267	
	TTF1	8	FFPE	3.750 + 1.195	0.0002
			PFPE	1.5 + 0.378	
	MNF116	8	FFPE	3.625 + 0.582	0.0005
			PFPE	4.625 + 0.231	
Prostate	p63	9	FFPE	4.5 + 0.25	0.6700
			PFPE	4.556 + 0.3	
	34BE12	9	FFPE	4.944 + 0.167	0.1334
			PFPE	4.667 + 0.5	
	CK5	9	FFPE	4.722 + 0.363	0.0018
			PFPE	4.056 + 0.391	
	p63/racemase	9	FFPE	4.056 + 0.3	0.7498
			PFPE	4.111 + 0.417	
	EGFR	6	FFPE	3.833 + 0.258	0.4334
			PFPE	3.714 + 0.267	
	p16	8	FFPE	3.750 + 0.463	0.7000
			PFPE	3.813 + 0.259	

Staining was blindly scored by two independent qualified observers. Data represents mean score + SD. A paired Student’s *t*-test was performed to determine significance

In colon samples from site 1, 4/7 (57%) of the antibodies tested, that is, nuclear markers MLH1, MSH2, MSH6 and PMS2, worked better in FFPE samples compared with PFPE samples, where there was less intense expression overall (*p* < 0.05). On the contrary, only one antibody (CK5) out of five tested in prostate samples performed better in FFPE samples compared with PFPE. For the other antibodies, the results were comparable between PFPE and FFPE samples, whether cytoplasmic, membranous or nuclear markers. Blind scoring of IHC performed following standard FFPE protocols in site 3 also revealed that in general the observers preferred FFPE samples, and there appeared to be a particular issue with less intense expression of nuclear proteins such as p63 and Ki67.

To optimise the staining originally set up for FFPE samples, a 24 h incubation in 10% formalin buffer was introduced before staining for some antibodies. In site 3, following pre-treatment in formalin, staining of three (Ki67, TTF1 and MNF116) out of four of these antibodies was significantly improved (analysis in Table 4).

**Table 4:** Comparison of IHC with and without pre-treatment

Antibody	Number of cases	PFPE no pre-treatment (mean + SD)	PFPE with pre-treatment (mean + SD)	No pre-treatment versus pre-treatment (*p*-value)	FFPE (mean + SD)	PFPE with pre-treatment versus FFPE (*p*-value)
P63	8	1.250 + 0.267	2.250 + 0.886	0.0086	3.438 + 1.613	0.0067
TTF1	8	1.5 + 0.378	2.938 + 0.320	0.0001	3.750 + 1.195	0.2216
MNF116	8	4.625 + 0.231	3.875 + 0.694	0.0117	3.625 + 0.582	0.3159
Ki67	4	2.875 + 1.732	2.875 + 1.041	0.5	4.250 + 1.443	0.2990

Pre-treatment, involving a 24-h incubation of slides in formalin prior to staining, was used to test its effectiveness in improving staining of four antibodies; p63, TTF1, MNF116 (all lung tissue) and Ki67 (three lymph tissue, one neuroendocrine). Slides were scored as described. Data represent mean score + SD. A paired Student’s *t*-test was performed to determine significance

**Fig. 3:**
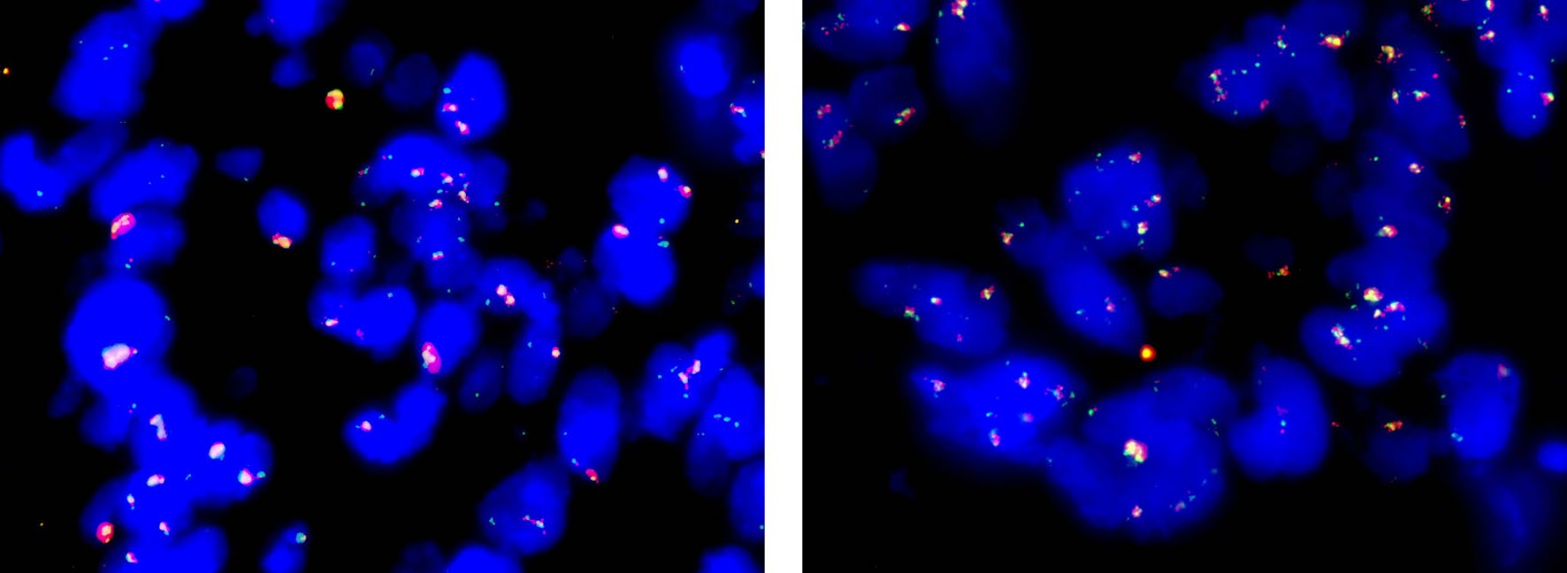
Representative images of FISH signals from EGFR probe. EGFR represented in red and centrometric enumeration probe (CEP) in green. Left PFPE, right FFPE

Regarding the FISH analysis, all FFPE cases and 37/40 (92.5%) PFPE cases were deemed adequate for diagnosis. The nuclear staining and the background did not differ between FFPE and PFPE samples. Epidermal growth factor receptor (EGFR) staining intensity was significantly higher in FFPE samples (3.9 + 0.308) compared with PFPE samples (3.175 + 0.467, *p* < 0.0001). CDKN2A staining intensity tended to be higher in PPFE samples compared with FFPE samples, but the difference was not statistically significant. While 18/20 FFPE (90%) cases had very strong signal for EGFR compared with only 6/20 (30%, *p* = 0.0001) PFPE samples, the results for CDKN2A were similar [FFPE 5/18 (27%) versus PPFE 6/18 (33%)]. Full scoring can be found in Table 5 and representative images in Fig. 3.

**Table 5:** FISH scoring of EGFR and CDKN2A probes

Tissue	Fixative	EGFR	*p*-Value	CDKN2A	*p*-Value	BCl2 (*n* = 6)	*p*-Value	BCL6 (*n* = 3)	*p*-Value
Colon (*n* = 10)	FFPE	3.80 + 0.42	0.0327	2.95 + 0.97	0.7	n/a	n/a	n/a	n/a
	PAXgene	3.30 + 0.54		3.10 + 0.99		n/a		n/a	n/a
Prostate (*n* = 8)	FFPE	4.00 + 0	0.0001	2.81 + 0.26	0.0001	n/a	n/a	n/a	n/a
	PAXgene	3.06 + 0.42		4.00 + 0		n/a		n/a	n/a
Lymph node	FFPE	4	n/a	4	n/a	2.58 + 0.38	1	2.66 + 0.57	0.4
	PAXgene	3		3		2.58 + 0.49		3 + 0	
Lung (*n* = 1)	FFPE	4	n/a	4	n/a	n/a	n/a	n/a	n/a
	PAXgene	3		3		n/a	n/a	n/a	n/a
Total	FFPE	3.9 + 0.31	0.0001	2.95 + 0.826	0.088				
	PAXgene	3.17 + 0.47		3.42 + 0.893					

Cases were scored as follows. Intensity: 0 (absent), 1 (very weak), 2 (weak), 3 (strong), 4 (very strong). BCl2 and Bcl6 scoring: 1 (poor), 2 (analysable); 3 (good). Data represent mean score + SD. A paired Student’s *t*-test was performed to determine significance. Background and counterstaining were also quantified, with no differences observed (data not shown). Objective magnification ×100

### Molecular analysis

**Fig. 4:**
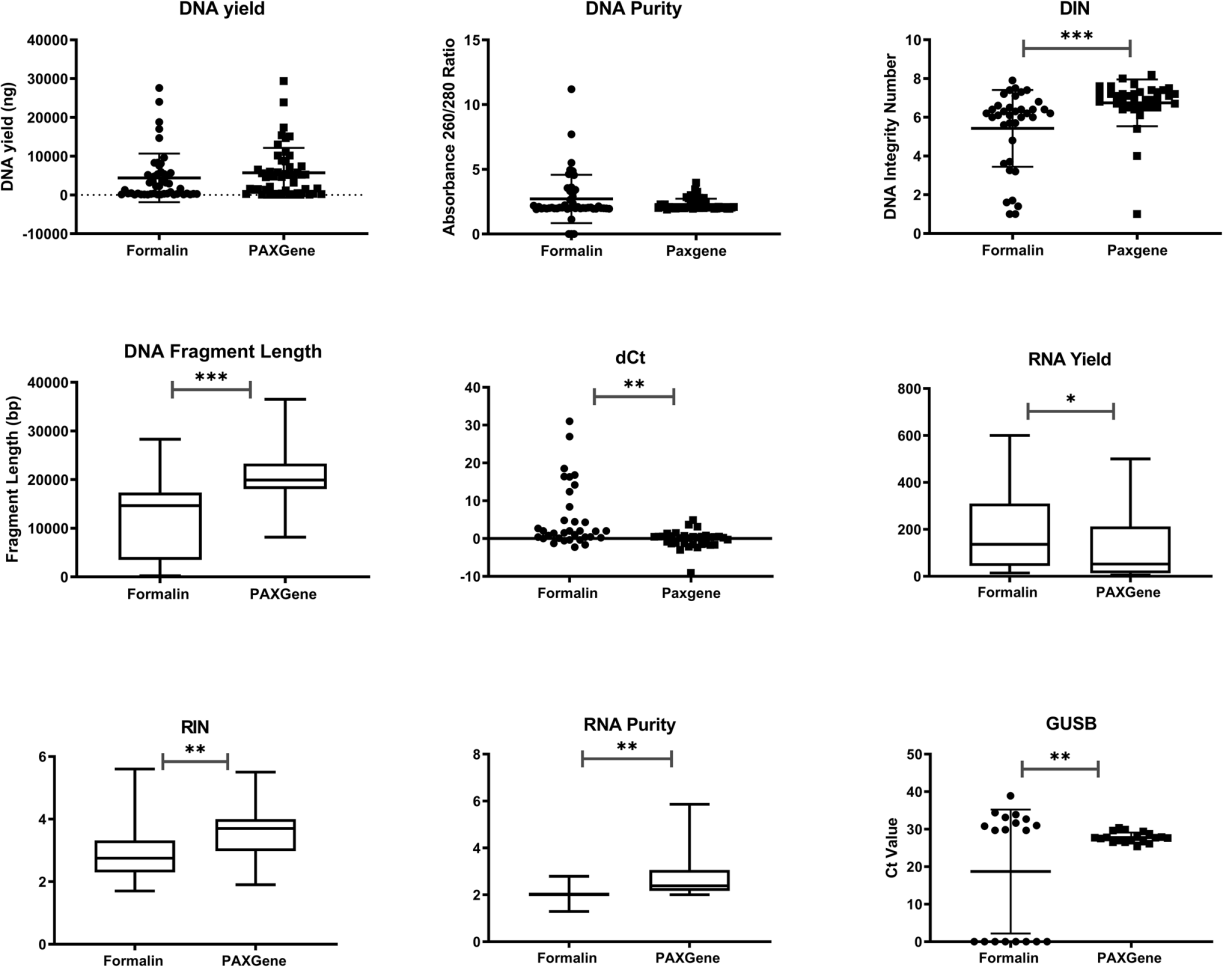
DNA and RNA assessment of quality. DNA and RNA obtained from paired formalin- or PAXgene-fixed samples were analysed using a number of methods to assess quality. No differences were observed by tissue type. Following exclusion of unquantifiable samples, the numbers of paired cases analysed were as follows: DNA yield (*n* = 56), DNA purity (*n* = 66), DIN (*n* = 44), DNA fragment length (*n* = 58), dCt (*n* = 39), RNA yield (*n* = 55), RIN (*n* = 55), RNA purity (*n* = 63), GUSB dCt (*n* = 19). Data represent mean score + SD. A paired Student’s *t*-test was performed to determine significance (*p < 0.05, **p < 0.01, ***p < 0.001). Details on how values were explained can be found in “Materials and methods”

The DNA yield from PAXgene-fixed samples was 4290.68 ng compared with 3888.82 ng in the FFPE samples, but the difference was not statistically significant (*p* = 0.7) (Fig. 4; Table 6). DNA fragment length and DNA integrity numbers were significantly higher in PFPE samples compared with FFPE (*p* < 0.001). Thirty-four paired samples were analysed by qPCR using the Infinium FFPE QC kit, and dCT values established using high-quality genomic DNA. Three samples failed to amplify (one FFPE and two PFPE). Mean dCT values were 7.98 (FFPE) and −0.58 (PFPE) (*p* < 0.01).

**Table 6:** DNA and RNA assessment

	Fixative	Mean read length bp	On target	Variants	
Sequencing	PFPE	122.5	0.9716	22	
	FFPE	118	0.97935	26.5	
	FF	120.5	0.9706	23	

		Yield (ng)	RIN	RNA purity	
RNA	FFPE	5130.33	2.8	2.02	
	PFPE	3112.78	3.9	2.55	
	*p*-Value	0.03	< 0.01	< 0.01	

		Yield (ng)	DNA fragment length (bp)	DIN	DNA purity
DNA	FFPE	3955.42	14,317.02	5.69	2.85
	PFPE	5153.29	21,452.93	6.77	2.63
	*p*-Value	0.31	< 0.001	< 0.001	0.52

Data represent mean scores of quality metrics used to assess DNA and RNA quality. DNA was also evaluated by next-generation sequencing quality metrics, compared with fresh frozen extracted DNA (*n* = 4). Following exclusion of unquantifiable samples, the numbers of paired cases analysed were as follows: DNA yield (*n* = 56), DNA purity (*n* = 66), DIN (*n* = 44), DNA fragment length (*n* = 58), dCt (*n* = 39), RNA yield (*n* = 55), RIN (*n* = 55), RNA purity (*n* = 63), GUSB dCt (*n* = 19)

Analysis across all tissue types revealed that the RNA yield was higher in FFPE samples (4404.2 ng) compared with PFPE samples (3010.3 ng), but the results were not statistically significant (*p* = 0.03) (Table 6). However, RIN numbers and RNA purity were significantly higher in PFPE samples (2.85 and 3.66, respectively) compared with FFPE samples (2.03 and 2.98, respectively, *p* < 0.01). qPCR was performed to measure GUSB expression in 19 paired cases; Ct means were 18.72 for FFPE cases compared with 27.5 for PFPE cases (*p* < 0.01) after exclusion of unamplified samples.

**Fig. 5:**
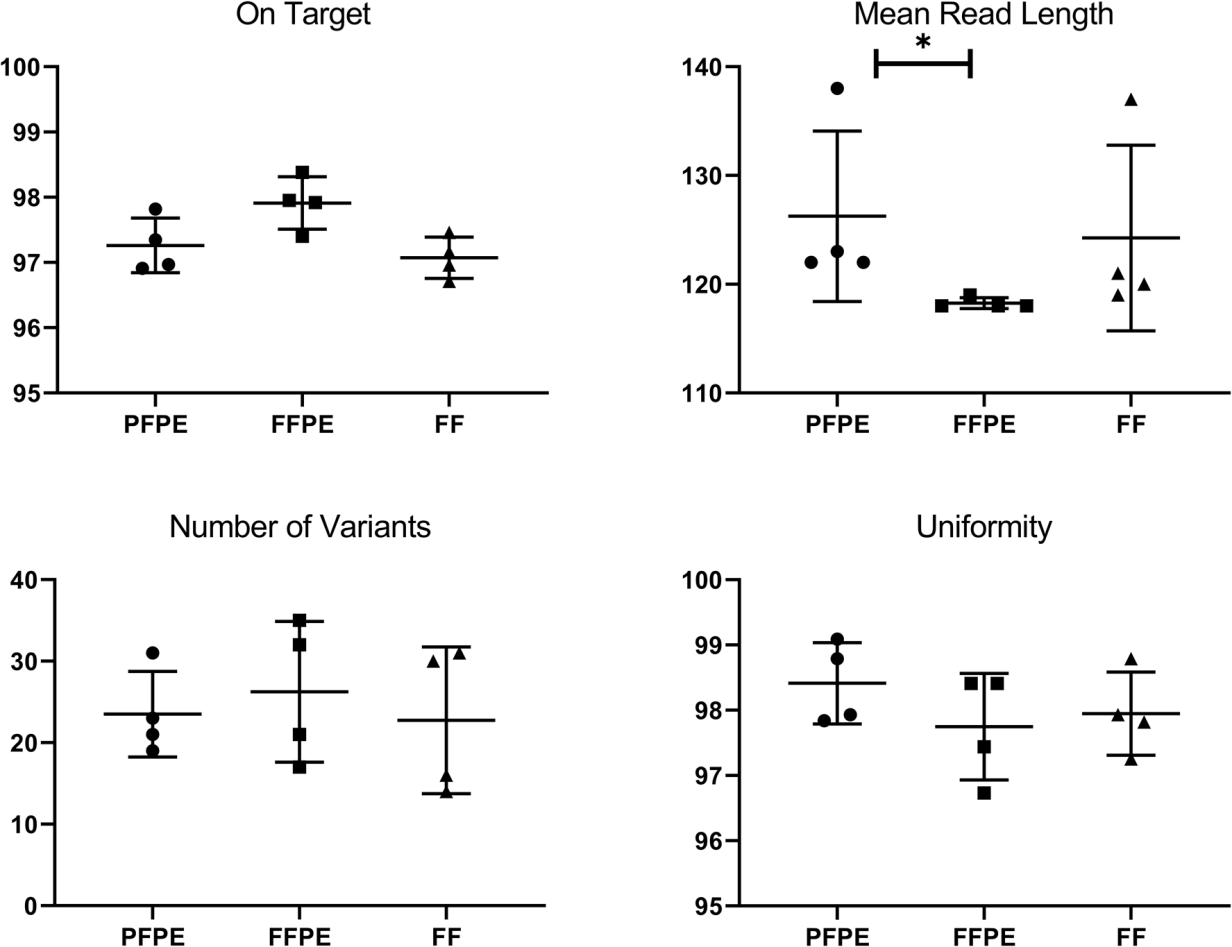
NGS quality metrics. NGS was performed on *n* = 4 triplets of DNA samples extracted from PFPE, FFPE or fresh frozen (FF) tissue. Data represent mean score + SD. Statistical significance was determined by Friedman test (*p < 0.05)

Regarding NGS QC metric data, only one significant difference was observed between FFPE and PFPE samples, whereby PFPE showed significantly longer mean read length (*p* < 0.05, *n* = 4, Friedman test) (Fig. 5). No other metrics showed significant improvement in quality, including the number of on target reads (median 97.93% versus 97.16%) or uniformity (median 97.93% versus 98.36%). The number of variants detected was increased in FFPE, although this was not significant (median 27 versus 22), indicative of artefact.

## Discussion

FFPE-based workflows are well established within pathology services and on the whole provide material suitable for current clinical diagnostic techniques. However, formalin fixation is damaging to biomolecules in the tissue and is potentially harmful to the user. For these reasons, there is an ongoing effort toward finding an alternative fixative. Here, we assess the potential replacement of formalin with PAXgene Tissue, an ethanol-based fixative designed to preserve biomolecules and integrate readily into current clinical workflows.

In terms of workflow, the PAXgene Tissue system was readily implemented in the participating departments. The requirement to transfer tissue from fixative to stabiliser solution was an additional step but was not time consuming or problematic with 5 days a week (Monday to Friday) sample collection and 6 days a week (Monday to Saturday) laboratory working. One important impracticality to note was the requirement of a dedicated tissue processor to maximise the benefit of formalin-free processing, an option that may not be available or cost efficient for all laboratories.

For microtomy, there were instances where PFPE tissue sections were slightly more difficult to produce due to cellular disaggregation and disintegration of necrotic tissue, and sections of affected cases often crumpled and creased on contact with the glass slide. In several cases, the tissue also floated off the slide during haematoxylin and eosin staining. While this was not a consistent occurrence, the likelihood of this problem could be reduced by ensuring the section was left to dry completely and using sticky (e.g. Super-Frost) slides. This exemplifies the likely requirement for subtle but important changes to the standard workflow for PFPE tissues.

Concerning the morphological analysis, blind scoring of H&E sections from paired formalin-fixed and PAXgene-fixed samples was comparable, with no significant overall difference in the scores. There was, however, a significant difference in scoring the cytoplasm, consistent with observations that PFPE sections stained more intensely with eosin in the cytoplasm (Kap et al. 2011). While the cytoplasmic staining was poorer in the PFPE-fixed tissues, the staining was still considered adequate for diagnostic purposes in most cases. This also suggest that PAXgene Tissue-fixed samples can be recognised easily in H&E-stained sections by pathologists, which therefore diminishes the power of the blind scoring.

These results are consistent with previously published data (Kap et al. 2011; Viertler et al. 2012; Belloni et al. 2013; Groelz et al. 2013). Kap et al. (2011) performed a comprehensive analysis of PFPE morphology on a range of normal tissue types. The group analysed 70 normal human tissue samples, and although scoring was not blinded, they deemed that differences between PFPE and FFPE tissues were minimal and that the elevated levels of eosinophilia noted in PFPE tissues did not seem to hinder observations. Similar results were reported using melanoma samples (Belloni et al. 2013) and also lung tumour tissue by another member of the STRATFix consortium (Southwood et al. 2020).

The morphological findings were different when the three sites were analysed separately. In general, site 1 and site 2 blinded observers preferred PFPE morphology over the FFPE samples while site 3 preferred FFPE samples. This difference could be explained by the experience of the observers and unconscious bias due to the scorers at one site having an awareness of the previously documented differences between FFPE and PFPE specimens on H&E. In site 1, the blind observers were involved in the project only for the slide evaluation, without background information regarding ongoing results or scope of the project. In contrast, in site 3, the observers were highly involved in the project management and data evaluation, potentially accounting for a more critical opinion of the material. Also, it is well recognised that there are differences in H&E appearances between laboratories in terms of staining intensity, leading to differing pathologist baseline expectations and preferences.

Immunohistochemical analysis revealed a generally lower staining intensity and performance in PFPE samples compared with FFPE samples. The results varied between the different sites and for tissue type. Well-established protocols such as MLH1, MSH2, MSH6, PMS2 and CK5 worked better in FFPE samples compared with PFPE samples (*p* < 0.05), while the other antibodies stained comparably between PFPE and FFPE samples, with no significant differences observed.

Previous work performed comparing FFPE and PFPE tissue for IHC staining has shown mixed results. Some publications reported that stained PFPE samples are interpretable but suboptimally immunostained (Belloni et al. 2013; Mathieson et al. 2016), while Southwood et al. (2020) found the immunostaining of PFPE and FFPE was similar, without tailoring optimisation or protocols.

In formalin fixation, cross-linking of proteins and disruption of hydrogen bonds both affect protein structure and, thus, binding of antibodies to epitopes. Furthermore, the subsequent processing of tissues through changes in temperature will cause proteins to fold and unfold, further altering the epitope, dependent on the cross-links formed during fixation. As a result, an antibody optimised for FFPE tissue will be specific for the epitope formed during formalin fixation and subsequent processing. Another obstacle is the antigen retrieval process, which is again optimised for FFPE tissue. It has been reported that PFPE tissue requires less stringent antigen retrieval (Kap et al. 2011; Stumptner et al. 2019), unsurprising since the epitope would be less altered.

This poses a challenge for PFPE, as IHC is an indispensable diagnostic technique and protocols used clinically are subject to stringent internal verification as well as the requirement for ongoing external quality assurance, especially for predictive markers (e.g. PD-L1; Dolled-Filhart et al. 2016; Roach et al. 2016). One aim of the PAXgene Tissue system, and of this work, is to integrate as effortlessly as possible into current infrastructure. For this reason, IHC protocols in this study were not initially optimised for PFPE tissue.

In an attempt to overcome suboptimal staining observed with some of the antibodies, staining was repeated by adding a 24-h incubation step with formalin for PFPE sections. Differences in the quality of staining were overcome with this pre-treatment step for three of four antibodies tested. While this establishes the importance of cross-linking for rescuing the correct protein epitope for FFPE-optimised antibodies, it also demonstrates that a *complete* replacement of FFPE tissue in clinical laboratories will require significant changes to current practices.

While PFPE samples are comparable histologically, the reported advantage of PAXgene fixation is better preservation of biological molecules (Groelz et al. 2013; Staff et al. 2013; Craft et al. 2014; Andersen et al. 2015; Gillard et al. 2015; Mathieson et al. 2016; Korenkova et al. 2016; Southwood et al. 2020). In line with existing reports (Groelz et al. 2013; Staff et al. 2013; Liu and Edward 2017), our RNA purity and RIN indicate PFPE to be superior to FFPE samples. We also found that DNA purity, integrity and fragment size were superior in PFPE specimens, again in keeping with previous studies (Andersen et al. 2015; Southwood et al. 2020). Our qPCR evaluation also supports these findings as we found that, on average, fewer PCR amplification cycles were required to exceed the background cycle threshold for PAXgene Tissue fixed samples, supporting superior biomolecule preservation.

Unlike other publications on the PAXgene Tissue system (Groelz et al. 2013; Staff et al. 2013), we did not obtain higher RNA yields from PFPE compared with FFPE specimens. While we cannot find a clear reason for this discrepancy, we are not the first PAXgene Tissue study where this is reported (Liu and Edward 2017). As this difference was not statistically significant and as our results were generally in line with recent reports on the better amplification performance of PFPE tissues, we did not investigate this matter any further.

NGS results were mostly comparable between FFPE, PFPE and fresh frozen samples. Fresh frozen samples are regarded to be superior in terms of preservation of nucleic acids, and thus the lack of significance between the tissue types is surprising. However, for the purposes of this study, both FFPE and PFPE samples were optimally preserved, avoiding over-fixation, and both paraffin blocks were kept at −20 °C, which is known to increase nucleic acid quality (Noguchi et al. 1997; Maraschin et al. 2017; Groelz et al. 2018; Schmeller et al. 2019). This could account for the improved performance of both types of fixed samples in this study.

## Conclusion

This analysis is based on the largest cohort of paired formalin and PAXgene Tissue fixed samples published to date, with data obtained from multiple established histopathology labs across the UK. The PAXgene Tissue system integrated readily into current infrastructure for sample collection and processing, and performed comparably on histological assessment of the tissue.

Further work would be required to determine its suitability for diagnostics, particularly where IHC is concerned, as many antibodies work optimally where formalin cross-links have formed between proteins. Crucially, we showed that IHC quality in PFPE tissues can be recovered with a formalin pre-treatment for TTF1, MNF116 and Ki67; however, further optimisations would be required for other staining protocols such as for P63. Although this seems a large undertaking, the optimisations would likely consist of minor changes within existing protocols, as demonstrated.

Additionally, the slight inferiority of morphology may be tolerable to many pathologists but is potentially problematic. It is possible to envisage a system where tissue can be preserved by both formalin and PAXgene where the morphology is especially sensitive to PAXgene fixation such as in the lymph nodes.

Overall, this study demonstrated, in a clinically representative context, that nucleic acid quality was improved when isolated from PFPE, while maintaining adequate histological staining for diagnostic purposes with minimal disruption to routine workflow. In a time where molecular diagnostics is becoming increasingly important, and we understand that the quality of biological molecules derived from FFPE tissues is unreliable and inconsistent, the PAXgene system offers an easily implemented solution to better preserving DNA while maintaining quality of the tissue for traditional diagnostic techniques.

## Supplementary Information

Below is the link to the electronic supplementary material.

Supplementary file1 (DOCX 16 KB)

Supplementary file2 (DOCX 108 KB)
