# Supplementary material for: Alternative tissue fixation for combined histopathological and molecular analysis in a clinically representative setting
Source: Histochem Cell Biol. 2021 Dec 14;156(6):595–607. doi: 10.1007/s00418-021-02029-1 (PMC8695534; doi:10.1007/s00418-021-02029-1)
Supplement: Supplementary file 1 — Supplementary file1 (DOCX 16 KB) [file 418_2021_2029_MOESM1_ESM.docx]

**Table S1. Details of antibodies used for IHC**

| **Antibody** | **Clone** | **Company** | **Cat Number** | **Platform** |
| --- | --- | --- | --- | --- |
| P63 | DAK-p63 | Dako | M7317 | Leica Bond III |
| HMWCK | 34BE12 | Dako | M0630 | Leica Bond III |
| CK5 | XM26 | Leica | NCL-L-CK5 | Leica Bond III |
| p63/racemase (AMACR) | 13H4 | Dako | M3616 | Leica Bond III |
| MLH1 | ES05 | Leica | NCL-L-MLH1 | Leica Bond III |
| MSH2 | FE11 | Dako | M3639 | Leica Bond III |
| PMS2 | A16-4 | Pharmingen | 556415 | Leica Bond III |
| MSH6 | EP49 | Dako | M3646 | Leica Bond III |
| p53 | DO7 | Leica | NCL-L-p53-DO7 | Leica Bond III |
| HER2 | 4B5 | Roche | 90-2991) | Ventana Benchmark Ultra |
| CD3 | LN10 | Novocastra | PA0553 | Leica Bond III |
| CD4 | 4B12 | Novocastra | PA0427 | Leica Bond III |
| CD20 | L26 | Novocastra | PA0200 | Leica Bond III |
| MIB-1 | Mib-1 | Dako |  | Leica Bond III |
| TTF1 | SPT24 | Novocastra |  | Leica Bond III |
| CK7 | RN7 | Novocastra |  | Leica Bond III |
| ALK1 | 5A4 | Novocastra |  | Leica Bond III |
| Chromogranin | 5H7 | Leica Microsystems | NCL-CHROM-430 | Dako AS48 |
| HMB45 | HMB-45 | Dako | M0634 | Dako AS48 |
| Melan A | A103 | Leica Microsystems | NCL-L-MELANA | Dako AS48 |
| MIB 1 | Ki-67 | Dako | M7240 | Dako AS48 |
| Cytokeratin MNF116 | MNF116 | Dako | M0821 | Dako AS48 |
| p63 | 7JUL | Leica Microsystems | NCL-p63 | Dako AS48 |
| Synaptophysin | 27G12 | Leica Microsystems | NCL-L-SYNAP-299 | Dako AS48 |
| S100 | Polyclonal | Dako | Z0311 | Dako AS48 |
| TdT | SEN28 | Leica Microsystems | NCL-L-TdT-339 | Dako AS48 |
| TTF1 | SPT24 | Leica Microsystems | NCL-L-TTF-1 | Dako AS48 |
| CD3 | LN10 | Leica Microsystems | NCL-L-CD3-565 | Dako AS48 |
| CD10 | 56C6 | Leica Microsystems | NCL-L-CD10-270 | Dako AS48 |
| CD15 (Leu M1) | MMA | Bd Biosciences | 347420 | Dako AS48 |
| CD20 | L26 | Dako | M0755 | Dako AS48 |
| CD30 | Ber-H2 | Dako | M0751 | Dako AS48 |
| CD56 (NCAM) | CD564 | Leica Microsystems | NCL-CD56-1B6 | Dako AS48 |

Platforms as follows: Dako AS 48 – Dako Link Autostainer 48, Dako, Cambridge, UK, with pre-treatment for formalin-fixed paraffin-embedded tissue using heat and a high pH buffer (heat-induced epitope retrieval, HIER) on the Dako PT Link and using the EnVision™ FLEX ready to use kit (Code GV925, Agilent, Stockport, UK); Leica Bond III—Leica Biosystems, Cambridge, UK using the Leica Bond Polymer Refine (DAB) detection system (Cat. DS9800, Leica Biosystems); Ventana Benchmark Ultra – Roche Diagnostics, Burgess Hill, UK using the Ventana UltraView DAB detection system (Cat. 760–500, Roche Diagnostics).
