# Supplementary material for: Alternative tissue fixation for combined histopathological and molecular analysis in a clinically representative setting
Source: Histochem Cell Biol. 2021 Dec 14;156(6):595–607. doi: 10.1007/s00418-021-02029-1 (PMC8695534; doi:10.1007/s00418-021-02029-1)

**Table S2. H&E Slide Scoring analysed by site and tissue type.**

Table shows comparison of blinded scoring of H&amp;E sections across the 3 sites where samples were collected, and also scoring by tissue type. Data represents the mean + SD for each tissue group, for membrane, nuclear and cytoplasmic staining. A paired Student <Emphasis Type="Italic">t</Emphasis> test was performed to determine significance.
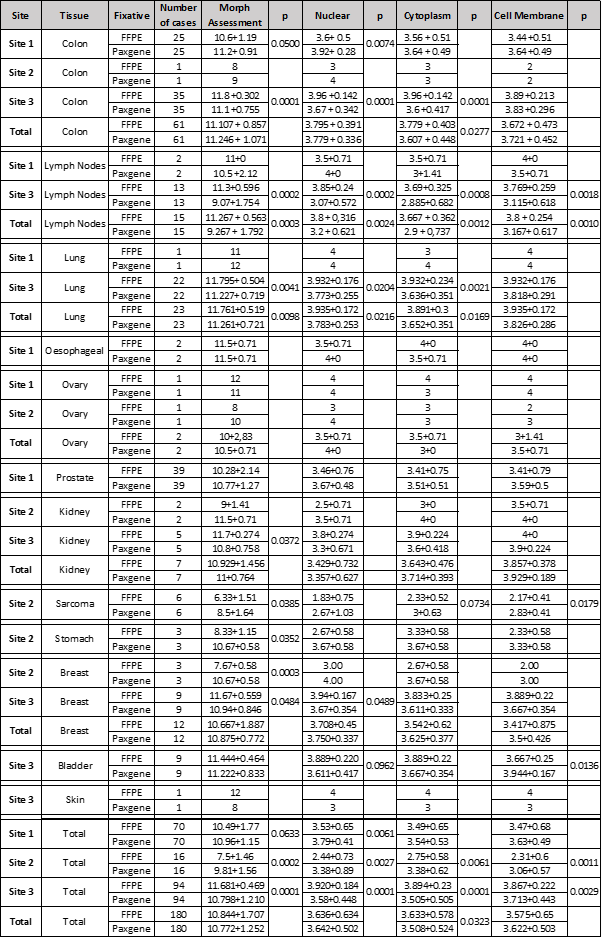

Supplement: Supplementary file 2 — Supplementary file2 (DOCX 108 KB) [file 418_2021_2029_MOESM2_ESM.docx]
